# Polymerization and Structure of Opposing Polymer Brushes Studied by Computer Simulations

**DOI:** 10.3390/polym13244294

**Published:** 2021-12-08

**Authors:** Krzysztof Halagan, Michal Banaszak, Jaroslaw Jung, Piotr Polanowski, Andrzej Sikorski

**Affiliations:** 1 Department of Molecular Physics, Faculty of Chemistry, Lodz University of Technology, Żeromskiego 116, 90924 Lodz, Poland; jaroslaw.jung@p.lodz.pl (J.J.); piotr.polanowski@p.lodz.pl (P.P.); 2 Faculty of Physics, Adam Mickiewicz University, ul. Uniwersytetu Poznańskiego 2, 61614 Poznań, Poland; mbanasz@amu.edu.pl; 3 NanoBiomedical Centre, Adam Mickiewicz University, ul. Wszechnicy Piastowskiej 3, 61614 Poznań, Poland; 4 Faculty of Chemistry, University of Warsaw, Pasteura 1, 02093 Warsaw, Poland; sikorski@chem.uw.edu.pl

**Keywords:** dynamic lattice liquid model, Monte Carlo method, polymer brushes, polymerization

## Abstract

A model of the polymerization process during the formation of a pair of polymer brushes was designed and investigated. The obtained system consisted of two impenetrable parallel surfaces with the same number of chains grafted on both surfaces. Coarse-grained chains embedded in nodes of a face-centered cubic lattice with excluded volume interactions were obtained by a ‘grafted from’ procedure. The structure of synthesized macromolecular systems was also studied. Monte Carlo simulations using the dynamic lattice liquid model were employed using dedicated parallel machine ARUZ in a large size and time scale. The parameters of the polymerization process were found to be crucial for the proper structure of the brush. It was found that for high grafting densities, chains were increasingly compressed, and there is surprisingly little interpenetration of chains from opposite surfaces. It was predicted and confirmed that in a polydisperse sample, the longer chains have unique configurations consisting of a stretched stem and a coiled crown.

## 1. Introduction

Polymer brushes are built from macromolecules terminally attached to a surface. They were recently a subject of many experimental and theoretical works predominantly because of their practical importance for, size-exclusion chromatography, polymer adhesion, lubrication and intelligent polymeric systems [1,2,3,4,5] and others. Polymer brushes formed of chains grafted to one surface can be treated as the reference state for the confined brushes. Brushes have been obtained using various experimental techniques, as recently reviewed [6,7,8]. Polymer brushes, in real experiments, can be synthesized using two different methods in general: by tethering the chains that were previously polymerized (‘grafting to’) and by growing chains directly from initiators anchored on the surface (‘grafting from’) [9,10,11,12].

Properties of polymer brushes were also studied using Molecular Dynamics, Dissipative Particle Dynamics [13,14,15,16,17], Monte Carlo (MC) simulations [14,18,19,20,21,22,23,24,25,26,27,28,29,30,31], scaling theory and theoretical self-consistent field considerations [31,32,33,34,35,36,37,38,39]. Field theory is particularly well suited to handle polymer brushes—a configuration of a single chain can be mapped into a trajectory of quantum particles with time being replaced by the contour length [34,35,36,37,38,39,40]. Although Milner, Witten, and Cates analytically employed a parabolic form for the concentration profile, more detailed approaches required numerical calculations [34,41]. Nevertheless, this theory can give an intriguing glimpse into the behavior of polymer brushes without performing time-consuming Monte Carlo simulations. More recently, polymer brushes with high grafting densities have been shown to be much more stretched, as was previously estimated [42]. The concentration profiles of a polymer brush swollen in good solvents have been extensively reported, for example, by Murat and Grest [16], where a molecular dynamics study was presented, and the results compared favorably with the monomer concentration profile obtained by field theory. In particular, their numerical result for intermediate surface coverage values agreed well with the analytic result obtained by Milner et al. [34] but differed qualitatively from it for higher surface coverage values. Other structural parameters mainly concern the chain size, its components parallel and perpendicular to the grafting surface, as well as the height of the brush [5,15,43].

Systems consisting of two opposing brushes, i.e., two parallel surfaces grafted with chains, were also a subject of considerable interest [44]. Theoretical studies mainly concerned the compression phenomenon of such brushes [45,46,47,48,49] by mutual interaction. Structure, interaction, and friction between a pair of brushes (neutral and charged) were recently investigated [47,50,51,52,53,54,55,56] using computer simulations. In a recent simulation study based on the dynamic lattice liquid (DLL) model, the attention was focused on the dynamics of dense and interpenetrating brushes [57]. It was shown that there was a strong correlation between the dynamics and the brush structure, and that changes in the mobility of solvent molecules could be attributed to the local structure of the brush.

In this paper, a coarse-grained MC model of opposing brushes, formed by multichain polymer systems, was considered. Due to complex architecture resulting from large size and high density of polymer chains, these systems were studied by a lattice model. Fully flexible chains immersed in a good solvent were considered, and solvent molecules were explicitly included in the model. At first, a polymerization process of opposing polymer brushes was studied. Brushes were virtually synthesized by ‘grafting from’ approach; that is, the chains’ polymerization started from the initiator present in the surfaces. In the ‘grafting from’ procedure, the initialization of the polymerization process starts on a surface and all chains grow until the process is terminated. This procedure was chosen due to the severe problems with the equilibration of brushes obtained by the ‘grafting to’ procedure, where completely synthesized chains are grafted onto surfaces. The properties of this model were studied using the Monte Carlo method using the DLL model [58,59]. This simulation algorithm was already successfully used, for example, to study various polymerization processes, including single polymer brushes [60,61,62]. The influence of the polymerization parameters on the structure of the growing opposing brushes was the main goal of this study. Then, the structure was investigated, focusing on the interactions of a pair of brushes.

## 2. The Model and Method

The model presented in this paper was coarse-grained, i.e., it consisted of coarse-grained fragments of matter such as polymers, polymer segments, and solvent molecules with all atomic details suppressed. The DLL model is based on the concept of cooperative motion of objects with positions restricted to nodes of a quasi-crystalline lattice. In the present study, it was a face-centered cubic lattice with coordination number *q* = 12. A small excess volume is assumed in the system, and therefore all objects have a space to vibrate around their position as in real dense liquids [58]. The quasi-crystalline lattice nodes define the positions of objects. Due to high density (all neighboring lattice nodes are occupied), an object is not able to easily move over a distance longer than lattice constant, but long-range motion can take place, in a long time limit, as a set of cooperative rearrangements. In the DLL model, the cooperative motion of objects has the form of a closed loop of displacements involving at least three neighboring objects in a given time step. Therefore, contrary to other lattice models, DLL allows studying systems at the highest density, where all lattice nodes of the system are occupied.

The DLL model was implemented as a Monte Carlo dynamic simulation algorithm for polymer brushes in a solvent. In the presented model, during the first step (the polymerization process), the lattice nodes are occupied either by surface nodes (some of them with initiating sites), segments of the polymer chains or monomers (free segments), whereas during the second step (the time evolution of the synthetized opposing brush), only polymer segments and solvent molecules are present (except the surfaces). The system was assumed to be athermal, i.e., without any interactions, with the exception of the excluded volume condition. Therefore, the polymerization and time evolution of opposing brushes were studied under good solvent conditions. The single simulation step *t* consisted of three stages. In the first one, a random vector field of motion attempt was generated. This was realized by assigning a unit vector to each object, pointed towards one of the nearest neighboring lattice sites and representing the direction along which the object attempted to move. In the second stage, groups of vectors forming closed loops were identified, whereas the remaining objects were immobilized at the given time step. To maintain the continuity of macromolecules, if the potential movement realized by a given loop led to a break of bond between chain segments in the polymer, such a loop was immobilized. In the third stage, rearrangements of objects along remaining loops were performed by displacing the objects to the neighboring lattice nodes according to the unit vector generated in the first stage. As a result, the time step was a discrete variable for which the positions of all objects were attempted to update simultaneously. A single time step in the DLL model corresponds to approximately 6 × 10^−13^ s or to 3 × 10^−12^ s for low-weight and high-weight macromolecular systems, respectively [26,63]. Detailed balance and ergodicity of the DLL algorithm was discussed elsewhere [58].

The simulation procedure consisted of two parts. In the first one, chains grafted onto a pair of parallel surfaces were synthesized using the ‘grafted from’ procedure. The procedure used enabled the obtainment of a polydisperse, dense and highly grafted opposing polymer brush [7,26]. Then, depending on the parameters of the polymerization process, equilibration runs were performed (see the discussion below). In the second part, for a polymer brush at equilibrium, a long production run delivered data for structure studies.

## 3. Results and Discussion

The system has a form of a slit built by a pair of parallel surfaces placed at *z* = 1 and 144 coordinates, the slit width was 2*d* = 142. The confining surfaces were impenetrable for monomers, polymer segments and solvent molecules. No other interactions with the surfaces were assumed. The length of the Monte Carlo box in directions parallel to the surfaces was *L* = 144 (72 nodes in *x*- and 144 nodes in *y*-direction), thus the system consisted of total 1,492,992 lattice nodes. Periodic boundary conditions were imposed in *x*- and *y*-directions. After polymerization, the end of each chain was grafted (tethered) to one of the surfaces and the positions of the grafting points were random. Both surfaces had the same amount of polymer chains.

Grafting density was defined as a ration of the grafting points number to the number of lattice nodes forming the surfaces. In this work, the grafting density was varied: *σ* = 0.2, 0.25, 0.3, 0.35, and 0.4 (for each surface). System contained up to 8200 polymer chains (*L*^2^*σ*). The critical grafting density σ* is usually defined as [15] σ* = π *R_g_*^2^*N*/*L*^2^, where *R_g_*^2^ is the mean-squared radius of gyration for chain and *N* is the number of chains grafted onto the surface. σ* measures the compression of grafted chains: for σ* < 1 chains are in the so-called mushroom regime, whereas for σ* > 1 chains form a real brush (chains are restricted to less surface area than in unrestricted solution). In this work σ* varied from 62 to 149, so the chains considered were always in the real brush regime [4]. For length dependance studies *σ* = 0.3 was selected. The influence of the grafting density on the single-brush structure showed that a crossover from low to high grafting regime is located near this value and it corresponds to ~0.35 chains/nm^2^ in a polymer system where one polymer bead represents the MMA monomer [26]. The synthesized systems always possessed some polydispersity and therefore the average chain length must be described by the averaged degree of polymerization. The number-averaged degree of polymerization *DP_n_* is defined as $DP_{n}=\sum_{i=1}^{2N}n_{i}m_{i}/\sum_{i=1}^{2N}n_{i}$, where 2*N* stands for the total number of chains in the system, *m_i_* is the length (number of segments) of the *i*-th chain, and *n_i_* is the number of chains with length *m_i_*. The *DP_n_* values were varied in a wide range, from 30 to 160.

**Figure 1:**
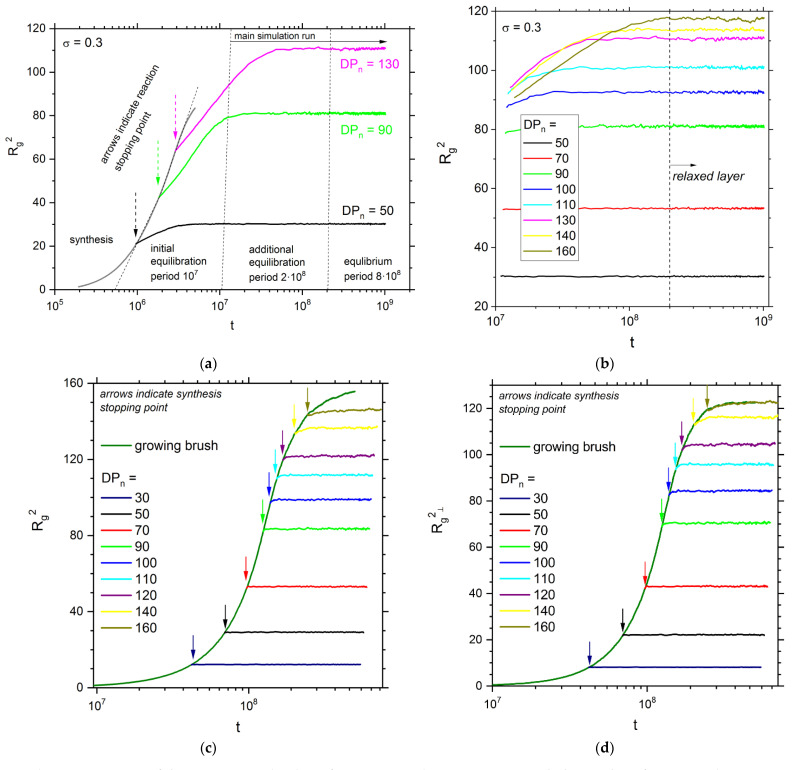
Variation of the mean squared radius of gyration, *R_g_*^2^ [lattice constants] with the number of Monte Carlo time steps *t* for: (**a**) three different degrees of polymerization with *p* = 10^−4^, (**b**) the equilibration and production run only, (**c**) the production run start marked with arrow in the case of *p* = 10^−6^, and (**d**) the changes of the normal component of the mean-squared radius of gyration <*R_g_*_⊥_^2^> during the production run.

The polymer brush system was synthesized in the first part of the simulation. The simulation box was filled with single-node monomer and initiator, which was randomly incorporated into the surfaces with a given grafting density. The controlled living irreversible radical polymerization was chosen for synthesis modeling, i.e., the process of attachment of monomers to a growing chain with assumed reaction rate *p*. In the first variant, the macromolecular layer was polymerized with *p* = 10^−4^ (see ref. [26,64] for details), and the brush grew until it reached the desired *DP_n_*. After this the reaction was stopped and all the unreacted monomer was replaced by an inert solvent. According to previous studies [26,64], a high polymerization probability, i.e., a fast polymerization, led to the more compact structures of polymer chains in a brush; therefore, the opposing brush systems obtained in this polymerization procedure required a long equilibration. Next, additional simulation steps t were performed to equilibrate the layer (10^7^ steps) because after the polymerization, chains needed extra time to extend to their equilibrium size [64]. The additional equilibration (2 × 10^8^ steps) was introduced for the reason discussed below. After the equilibration period, the production simulation run was begun (8 × 10^8^ steps). The second step allowed for good time-averaging of static properties, and determination of the system structure. Figure 1a presents examples of changes in the mean chain size (the mean squared radius of gyration) during a single simulation run for three average chain lengths. A higher target chain length resulted in considerably longer synthesis and equilibration times—the production run for longer chains (degree of polymerization *DP_n_* = 130) was almost an order of magnitude shorter than that for short chains (*DP_n_* = 50). Figure 1b presents changes in chain size in all opposing brushes under consideration, but the first 10^7^ steps of the simulation and synthesis part are not shown here. It can clearly be seen that the relaxation of systems with *DP_n_* > 70 is considerably longer and reaches 10^8^ steps. Therefore, the equilibration period had to be extended beyond the initially assumed 10^7^ steps. Nevertheless, all of the macromolecular systems under consideration were properly equilibrated. One can see that for longer chains, an additional equilibration is required in order to obtain a stable mean size of chains. Polymerization and equilibration had to last for 2 × 10^8^ time steps. To collect uncorrelated data, the production run lasted up to 10^9^ time steps. In order to study brushes in a wide range of grafting densities and chain lengths, one had to deal with systems consisting of 10^6^ objects (monomers, polymer segments and solvent molecules) or more. It appeared impossible to study such systems for at least 10^9^ time steps required for the equilibration process when using a typical computer cluster or supercomputer [65]. Therefore, the use of dedicated hardware like ARUZ (Analyzer of Real Complex Systems—in Polish, Analizator Rzeczywistych Układów Złożonych) is inevitable in order to study macromolecular systems at these time scales. The detailed information about this unique dedicated machine can be found elsewhere [57,65,66,67,68,69,70].

Having employed the ARUZ machine, an alternative way to equilibrate opposing brushes was examined. Basing on the results of our previous studies [26,64], *p* was lowered to the value of 10^−6^ (10^2^ times compared with the case discussed above), which is more realistic. It is impossible to relate this probability with polymerization reaction rates of given monomers but a proper ratio of this probability to the mobility of objects has to be set. The estimations suggest that for longer chains *p* should be no higher than 10^−5^. The relation of this parameter to experiments is discussed in ref. [26]. In Figure 1c, the time evolution of the mean squared radius of gyration of chains is presented for the probability *p* = 10^−6^. One can observe that after the polymerization was stopped, all *R_g_*^2^(t) curves are nearly flat. Additional information on the relaxation of polymer chains during the polymerization process can be obtained from the analysis of mean-squared radius of gyration component in the direction normal to the surface <*R_g_*^2^_⊥_>. This parameter is presented in Figure 1d as a function of time. The behavior of this parameter confirms that the system is at equilibrium: no additional changes in the direction perpendicular to the surface are observed. Thus, the obtained opposing brushes did not require any further equilibration after the synthesis, which confirms this choice of low reaction rate. The static properties of the brushes were time-averaged from 4 × 10^7^ time step for *DP_n_* < 120 and from 2 × 10^8^ for the remaining samples in order to minimize statistical errors. It appeared that the lowering of the polymerization probability also leads to smoother mass distributions and lower dispersity of chain lengths. The rest of presented results considers *p* = 10^−6^, if not stated otherwise, and presents the first clear indication that the formation of a dense polymer brush or an opposing brush system require a careful choice of simulation parameters. It also confirms the findings of dissipative particle dynamics simulations of triblock Janus particles, where the formation of assembled structures near surfaces required long simulation times [71,72].

**Figure 2:**
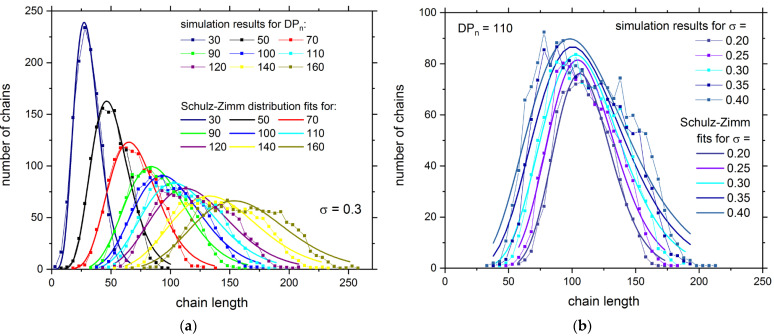
The distribution of chain lengths in opposing brushes for different degrees of polymerization *DP_n_* (**a**) and for different grafting densities *σ* (**b**). The points connected with thin lines represent Monte Carlo simulation results, whereas thick lines show the Schulz–Zimm approximations.

Figure 2 presents the distribution of chain lengths in both brushes for various *DP_n_* and *σ*. The results were averaged with the resolution of 5 polymer beads. It can be seen that each distribution has one peak and that the width of the peak increases with the increase in the *DP_n_* and the grafting densities. The increase in grafting density additionally slightly shifts the peak towards shorter chains and makes the distribution broader with data becoming more scattered. These distributions can be easily fitted with the two-parameter Schulz–Zimm function in a form already used for single brushes [73]. The Schulz–Zimm approximation works very well for all cases under consideration, contrary to results for single brushes where bimodal distributions were found for higher values of *DP_n_* [43,64]. These differences in mass distributions for opposing and single brushes have to be explained by lower polymerization probabilities used in the present study and thus confirm the proper choice of the reaction rate. The broadening of the mass distribution, i.e., existence of the significant number of longer chains, is apparently related to the presence of the other brush and its obstructive influence on the polymerization process. The second factor that leads to the formation of longer chains is the longer polymerization time, required for higher values of *DP_n_*.

**Figure 3:**
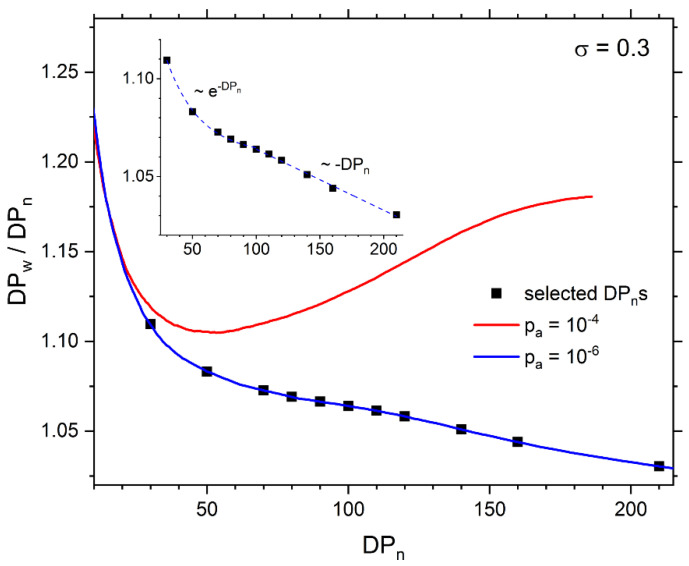
The dispersity *DP_w_*/*DP_n_* versus the number-averaged degree of polymerization *DP_n_*.

The distribution of chain lengths is usually analyzed via dispersity. Dispersity is defined as *DP_w_*/*DP_n_*, where *DP_w_* is the weight-averaged degree of polymerization defined as $DP_{w}=\sum_{i=1}^{2N}n_{i}^{2}m_{i}/\sum_{i=1}^{2N}n_{i}$. In Figure 3 the dispersity as a function of the number-averaged degree of polymerization *DP_n_* is shown. In the case of the polymerization probability *p* = 10^−4^, the behavior of dispersity is similar to the case of a single brush studied with the same model [26]: *DP_w_*/*DP_n_* initially decreases with *DP_n_*, but for higher values of *DP_n_* it does not reach a plateau and starts to increase. The crossover between two dispersity regimes is located near *DP_n_* = 50, i.e., where the brushes are still not influenced by each other. The reduction in the polymerization probability to the value of 10^−6^ dramatically changed the behavior of dispersity. It decreases with *DP_n_* over the entire range under consideration. This behavior is in opposite to the results obtained in Monte Carlo simulations where the bond fluctuation model was employed [27,28,29]. In these simulations, dispersity increases with the polymerization progress and with the number-average degrees of polymerization for most of grafting densities studied, that is, for *σ* > 0.08. This result is similar to the one presented herein obtained for a higher polymerization probability. Similar behavior of dispersity was found in Molecular Dynamics simulations of realistic models of oligomers grafted to surfaces and carbon nanotube composites [74,75].

**Figure 4:**
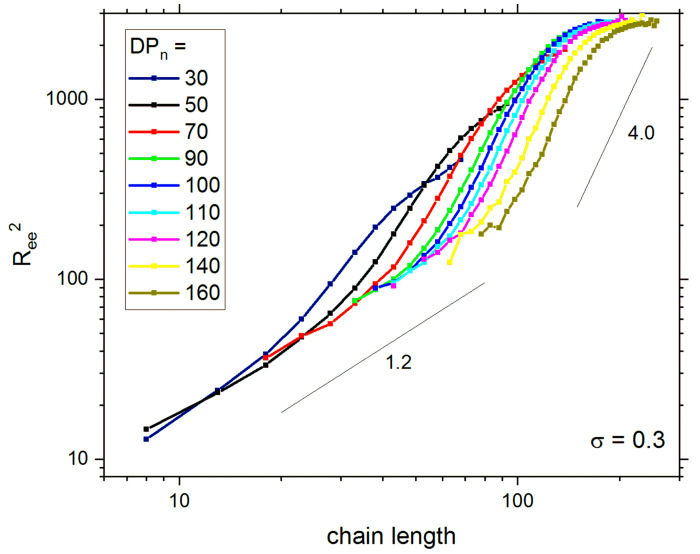
Mean squared end-to-end distance *R*^2^*_ee_* in lattice constants as a function of chain length *m*. The values of *DP_n_* are displayed in the inset.

The system structure in equilibrium can also be analyzed by the scaling behavior of chain sizes. Figure 4 shows the dependencies of the mean squared end-to-end distance *R*^2^*_ee_*, vs. chain length *m* (number of segments or *DP* for single chain)*,* and for various values of *DP_n_* on a double logarithmic scale. It is impossible to determine the scaling exponents because the plots presented in Figure 4 are not linear. One can recognize that for very short chains (*m* < 20) the scaling exponent is close to 1 whereas for intermediate lengths (50 < *m* < 100) the dependence of *R*^2^*_ee_* on chain length is considerably stronger. This unexpected scaling comes from a variety of states of shorter chains (coiled) and longer chains (extended). What is interesting here is that the size of the longest chains for high *DP_n_*, i.e., for systems where the pairs of brushes are in contact, is almost independent of the chain length. This confirms that the brushes do not interpenetrate significantly and that the compression effect for the brushes prevails. A detailed analysis of the dependency of the main parameters describing the brush on *DP_n_* was recently carried out [57]. It was shown that the mean chain height and radius of gyration exhibit two scaling regimes. The first region was found for brushes with *DP_n_* ≤ 100 and the scaling exponent was considerably higher than for single free chain [76] and very close to the exponent of rods. In the second regime, an unexpected scaling behavior was found, with scaling exponent below 1, which can be interpreted as a size increase for short chains with increasing *DP_n_* along with compression of longer chains, resulting in flower conformations. This suggested the mutual interaction of both brushes for systems with *DP_n_* > 100.

**Figure 5:**
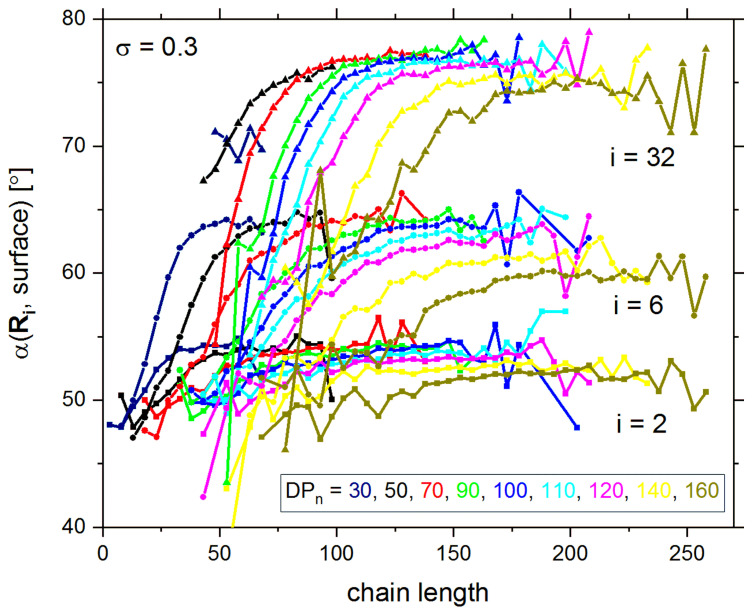
Angle between a vector from the grafting point and a polymer bead located in a given layer *i* and the grafting surface.

The main conclusions presented above, regarding the chain size parameters versus their length, can be supported by the chain orientations analysis. The angle *α* between the end-to-end vector *R_ee_* and the grafting surface (to which the chain was grafted) was calculated for this purpose [50]. It was recently shown that in polydisperse brushes, the orientation of short chains was almost random, whereas longer chains exhibit tilt angles near the value of 80°; that is, long chains are nearly perpendicular to the grafting surface. However, the longest chins are characterized by a slight decrease in tilt angles, apparently because of the excluded volume of the second brush. It is worth to notice that for longer chains (*m* > 200), there is no difference in tilt angles with respect to degree of polymerization *DP_n_,* although both brushes are being compressed [57]. Further information can be obtained from the analysis of angles formed between the grafting surface and the vector from the grafting point to a polymer bead belonging to the same chain and deposited in a layer number *I* (i.e., deposited at a distance *i* from the grafting surface), for example, *i* = 2 means coordinate *z* = 3. The results are presented in Figure 5, as a function of chain length, and suggest that in all layers, angles are always oriented more randomly for very short chains. For higher layers, the angle orientation is less random and closer to 80°. For longer chains (high *DP_n_*), the angles are lower. These differences are more pronounced for more distant layers, which confirms that the interpenetration is rather small and longer chains prefer to bend instead of penetrating the opposite brush. Summarizing, one can find in the system numerous relatively short chains and less populated longer chains. The latter are elongated in the direction normal to the grafting surface, whereas the short ones are placed more randomly, although the normal orientation prevails. Similar assumptions were made by Fleer et al. for their theoretical considerations [77], but it must be noted that the structure of the brush presented here was obtained directly from simulations. The increase in grafting density does not significantly change the shape of these curves, but shifts them toward lower values of chain lengths. The conformation of the described and discussed chain orientations can be found in the system visualization (see Figures 7 and 8).

**Figure 6:**
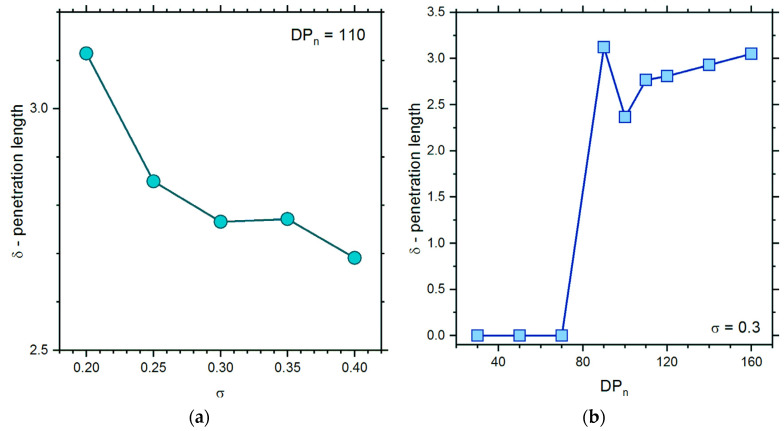
The interpenetration length *δ* as a function of the number-averaged degree of polymerization *DP_n_* (**a**) and of grafting density *σ* (**b**).

In order to check the state of mutual interpenetration of brushes directly, additional parameters were calculated. The overlap integral Γ, which is proportional to the number of brush-brush contacts per unit area, was defined according to the formula [39,78]: $\Gamma =\sum_{i=d}^{2d}\varphi_{1}\left(z_{i}\right)\varphi_{2}\left(z_{i}\right)$, where *ϕ*_1_ and *ϕ*_2_ are density profiles of each brush separately. The overlap integral is proportional to the number of interactions between polymer beads belonging to different brushes (in the model presented, there is no energy associated with these contacts—the system is athermal). Then, the mutual interactions of a pair of brushes can be described with the penetration length *δ* defined as $\delta =2\Gamma /\varphi_{tot}^{2}$, where *ϕ*^2^*_tot_* is the total density profile. Figure 6a presents the penetration length *δ* versus the number-averaged degree of polymerization *DP_n_*. The penetration length is zero for separated brushes, but between *DP_n_* = 70 and 90, where the brushes start to interact, this parameter increases rather weakly with the increase in *DP_n_* from ca. 2.5 to ca. 3. Theoretical predictions for monodisperse brushes gave a scaling *δ*~*m*^2/3^. For polydisperse systems, a larger interpenetration was expected, and the scaling depends on the degree of polydispersity: *δ*~*m*^1/3^ in the case of weakly polydisperse systems and *δ*~*m*^1/6^ for strongly polydisperse systems [45]. Thus, one can conclude that for dense opposing brushes at high grafting density, simple scaling theories do not work. Figure 6b shows the changes in the overlap integral with the grafting density. One can observe that the interpenetration of brushes is small and decreases over the entire range of grafting densities.

**Figure 7:**
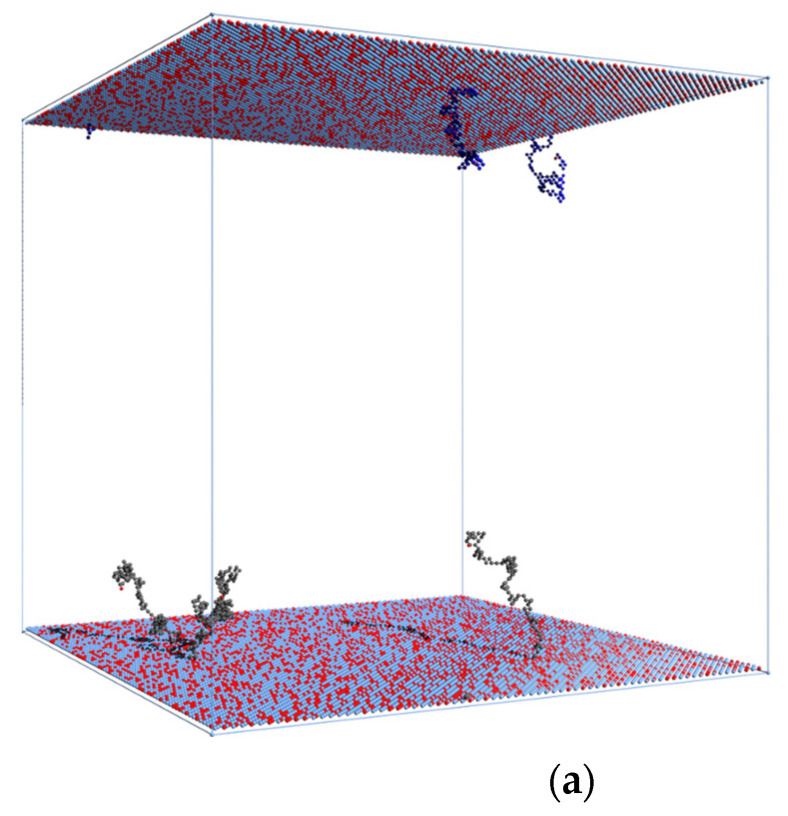

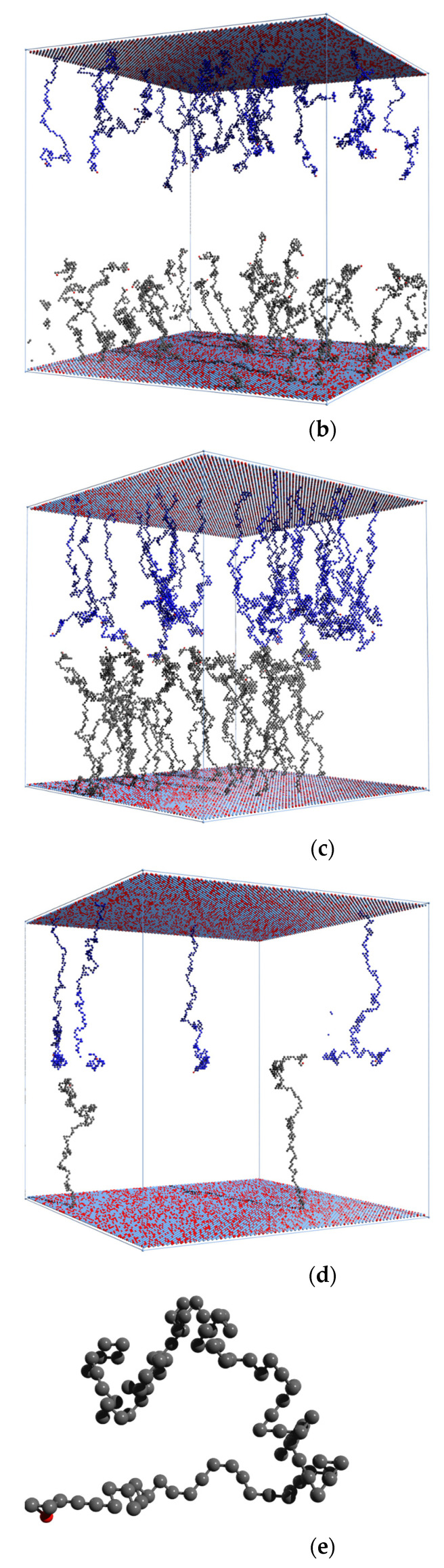

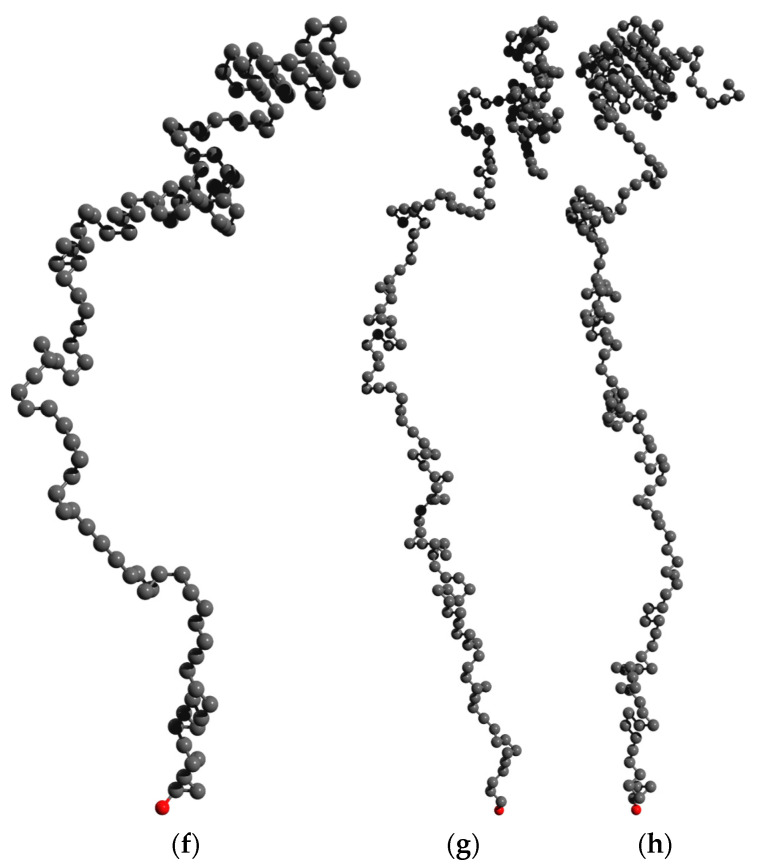
Typical chain conformations in the system with *DP_n_* = 160: *m* = 90 (**a**), *m* = 130 (**b**), *m* = 180 (**c**), and *m* = 230 (**d**). Enlarged typical single-chain conformations are also presented: (**e**) for (**a**), (**f**) for (**b**), (**g**) for (**c**), and (**h**) for (**d**).

**Figure 8:**
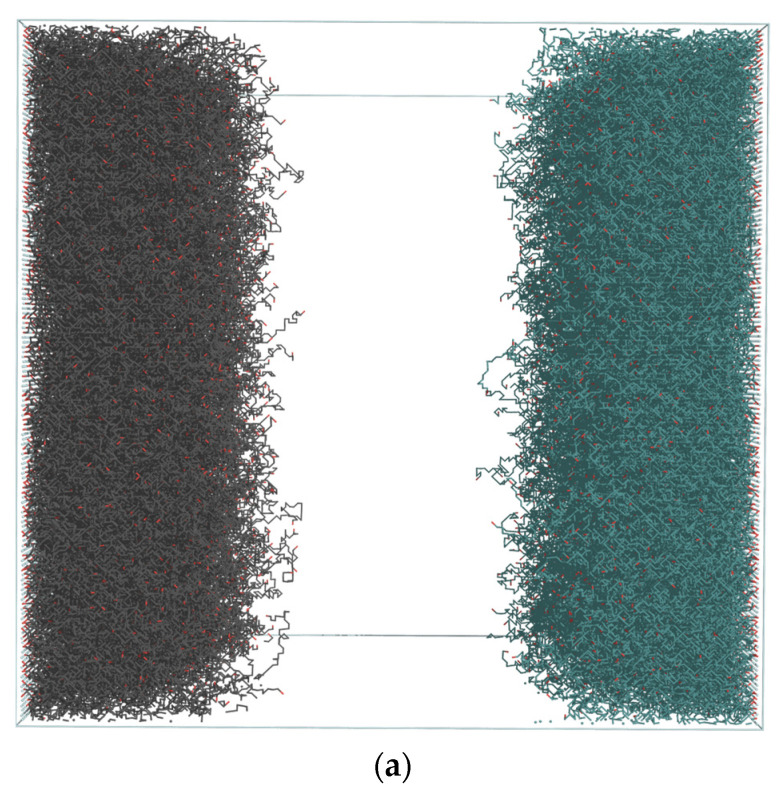

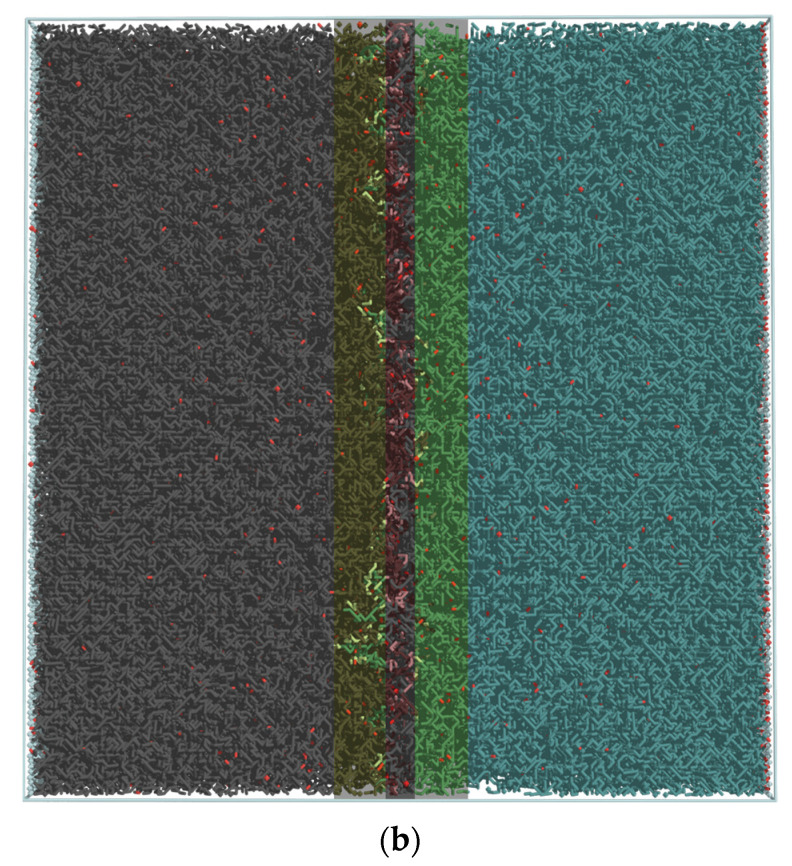
Configurations of an opposing brush system for *σ* = 0.3, *DP_n_* = 50 (**a**) and *DP_n_* = 110 (**b**). Each brush is displayed in a different color. Red dots mark the ends of the polymer chains. In (**b**) the penetration length (red bar) and density overlapping region (yellow bar) are indicated. Light grey chains are indicated as having a direct contact with opposing layer.

Figure 7 presents typical chain conformations in opposing brush systems for the highest number-averaged degree of polymerization *DP_n_* = 160. Short chains (*m* = 90, Figure 7a) are coiled, those of intermediate length (*m* = 130, Figure 7b) are stretched in the direction perpendicular to the surface, whereas longer chains (*m* = 180 and 230, Figure 7c,d) are also stretched in the same direction but are folded near their free ends. Partially folded states of longer chains confirm that the interpenetration of brushes is weak. The conformations shown in Figure 7 are in full agreement with the changes of the size parameters and chain orientations presented in Figure 4 and Figure 5, respectively, and discussed above. Thus, the presence of short and coiled chains near the grafting surface and long chains exhibiting conformations consisted of a stem and a crown predicted by theoretical considerations [74] was confirmed. In Figure 8a,b system snapshots are presented for *DP_n_* = 50 and 110, excluding solvent molecules for clarity. Each brush is marked with a different color to easily presenting the border between them. An increase in chain lengths leads to very weak brushes interpenetration. A similar observation was made for grafting density above *σ* = 0.3.

## 4. Conclusions

The polymerization process and structure of the opposing polymer brushes were studied using the dynamic lattice liquid (DLL) model, a unique Monte Carlo method based on the concept of cooperative motion. A dedicated hardware, i.e., Analyzer of Real Complex Systems (ARUZ), was also employed for these studies. The polymerization process for brushes build stage was performed with realistic parameters. The presented study provided suggestions concerning the parameters of the polymerization of brushes and opposing brushes. It was shown that a low polymerization probability (as in ‘real-life’ polymerization) can lead to equilibrium configurations of opposing brushes and no further equilibration was required. Moreover, proper mass distributions and dispersity were also reached in the polymerization process. Therefore, the results are relevant to real opposing brushes. Chains grafted to both surfaces were polydisperse (degrees of polymerization with a considerable dispersity) and distinguishable from those that were almost unconstrained (low degree of polymerization) and those that were highly compressed (high degree of polymerization). This allows for monitoring the structure as the system was being compressed.

The main conclusions are related to synthesis conditions of opposing brushes. It was recognized that the chain configurations obtained from fast polymerizations were significantly different from those obtained after equilibration during long polymerization. This put into question some of the Monte Carlo methods, such as the enrichment method: Ohno et al. [79] simply grew chains using this method, but the profiles and, therefore, also individual chains changed considerably after a long equilibration, as presented here. The presented results also showed that the density profiles of unconstrained and weekly constrained opposing brushes were nearly linear functions of the distance (except the edges) from the surfaces. This kind of behavior was expected for polydisperse brushes and, therefore, justifies the choice of simulation model.

It was also found that, as the chains were increasingly compressed (by employing longer chains), there was surprisingly little interpenetration of chains from opposite surface. This is not an obvious finding, although one has to remember that the investigated grafting density was high. The previous results obtained from the SCFT calculations [77] were confirmed. The unique configuration predicted and described as a flower conformation, consisting of a stretched stem and a coiled crown [77], was observed in a polydisperse sample for longer chains. It was shown that the short chains were oriented in directions mostly parallel to the grafting surfaces, whereas the long ones were perpendicular to the surfaces. Convincing arguments have been provided that the results presented herein are superior to SCFT which do not take into account fluctuations (due to the mean-field nature of SCFT) and those MC simulations that do not equilibrate the system properly or do not equilibrate it at all (such as the enrichment method).

## Data Availability

The data that support the findings of this study are available from the corresponding author upon reasonable request.
